# Multi-modal assessment of a cardiac stem cell therapy reveals distinct modulation of regional scar properties

**DOI:** 10.1186/s12967-024-04986-2

**Published:** 2024-02-21

**Authors:** Moritz Schweins, Ralf Gäbel, Matti Raitza, Praveen Vasudevan, Heiko Lemcke, Markus Joksch, Anna Schildt, Jens Kurth, Tobias Lindner, Felix G. Meinel, Alper Öner, Hüseyin Ince, Brigitte Vollmar, Bernd Joachim Krause, Robert David, Cajetan Immanuel Lang

**Affiliations:** 1 Department of Cardiac Surgery, Rostock University Medical Centre, 18057 Rostock, Germany; 2 Department of Life, Light and Matter, University of Rostock, 18059 Rostock, Germany; 3 Rudolf-Zenker-Institute for Experimental Surgery, Rostock University Medical Centre, 18057 Rostock, Germany; 4 Department of Nuclear Medicine, Rostock University Medical Centre, 18057 Rostock, Germany; 5 Core Facility Multimodal Small Animal Imaging, Rostock University Medical Centre, 18057 Rostock, Germany; 6 Institute of Diagnostic and Interventional Radiology, Rostock University Medical Center, Rostock, Germany; 7 Department of Cardiology, Rostock University Medical Centre, 18057 Rostock, Germany

**Keywords:** Mouse model, Myocardial infarction, Cell therapy, Regional wall analysis

## Abstract

**Background:**

The initial idea of functional tissue replacement has shifted to the concept that injected cells positively modulate myocardial healing by a non-specific immune response of the transplanted cells within the target tissue. This alleged local modification of the scar requires assessment of regional properties of the left ventricular wall in addition to commonly applied measures of global morphological and functional parameters. Hence, we aimed at investigating the effect of cardiac cell therapy with cardiovascular progenitor cells, so-called cardiac induced cells, on both global and regional properties of the left ventricle by a multimodal imaging approach in a mouse model.

**Methods:**

Myocardial infarction was induced in mice by ligation of the left anterior descending artery, the therapy group received an intramyocardial injection of 1 × 10^6^ cardiac induced cells suspended in matrigel, the control group received matrigel only. [^18^F]FDG positron emission tomography imaging was performed after 17 days, to assess regional glucose metabolism. Three weeks after myocardial infarction, cardiac magnetic resonance imaging was performed for morphological and functional assessment of the left ventricle. Following these measurements, hearts were excised for histological examinations.

**Results:**

Cell therapy had no significant effect on global morphological parameters. Similarly, there was no difference in scar size and capillary density between therapy and control group. However, there was a significant improvement in contractile function of the left ventricle – left ventricular ejection fraction, stroke volume and cardiac output. Regional analysis of the left ventricle identified changes of wall properties in the scar area as the putative mechanism. Cell therapy reduced the thinning of the scar and significantly improved its radial contractility. Furthermore, the metabolic defect, assessed by [^18^F]FDG, was significantly reduced by the cell therapy.

**Conclusion:**

Our data support the relevance of extending the assessment of global left ventricular parameters by a structured regional wall analysis for the evaluation of therapies targeting at modulation of healing myocardium. This approach will enable a deeper understanding of mechanisms underlying the effect of experimental regenerative therapies, thus paving the way for a successful translation into clinical application.

## Introduction

Hundreds of studies have been performed on cardiac stem cell therapies aiming at reducing myocardial damage after ischemic injury. Preclinical studies have consistently reported significant improvements of left ventricular ejection fraction (LVEF) following intramyocardial injection of different cell preparations since the very beginning [1]. Yet, the field is hampered by lacking success in translating this concept to patients and currently in a process of course correction as the concept underlying the mechanisms of cardiac cell therapies is rapidly changing. The idea of functional tissue replacement has been abandoned by most researchers and replaced by the concept of the so called “paracrine effects” mediated by the transplanted cells [2]. The latter has recently been challenged likewise, by proposing the hypothesis that injected cells exert their therapeutic effect merely by inducing an unspecific local immune response [3, 4]. Yet, particularly second-generation cardiac cell therapies, such as pluripotent stem cell derived cardiovascular cells, consistently show excellent therapeutic results in preclinical studies [5].

In contrast to a rapid evolution of molecular and cellular biology methods used in the majority of mouse studies, most researchers used a relatively rudimentary imaging approach without any information on regional wall properties [5]. Evidence from clinical studies suggests that global measures of left ventricular(LV) function are inappropriate for detecting the relatively small and regional effects of cardiac cell therapies [6]. Hence, integration of detailed local measures of cardiac function has been proposed to unravel the working mechanisms of cardiac cell therapies as early as a one decade ago. Dedicated small animal cardiac magnetic resonance imaging (CMR) provides high-resolution *in-vivo* assessment of cardiac function and morphology, but often requires elaborate and expensive software and profound expertise in CMR radiology. As a consequence, we have recently introduced a straight-forward tool in order to assess regional properties of the LV scar based on cine CMR [7]. In the current study, we use this protocol, to determine the local effects on the scar properties by intramyocardial injection of a cardiac stem cell product. The therapeutic cell preparation we used consists of embryoid bodies (EB) committed to the cardiac lineage, which we have introduced and characterized recently [8]. Using this approach, we show that the improvement of cardiac function is caused by a modulation of regional scar properties.

## Materials and methods

### Stem cell culture and cardiovascular differentiation

According to standard protocols W4 murine embryonic stem cells (mESCs), originally isolated from the 129S6 mouse strain, were grown as described previously [9]. In brief, cells were cultured in DMEM supplemented with 15% FBS Superior (Biochrom AG, Berlin, Germany), 1% Cell Shield® (Minerva Biolabs GmbH, Berlin, Germany), 100 µM non-essential amino acids, 1000 U/mL leukemia inhibitory factor (Phoenix Europe GmbH, Mannheim, Germany) and 100 µM β-mercaptoethanol (Sigma-Aldrich GmbH, Steinheim, Germany) at 37 °C, 5% CO_2_, and 20% O_2_. We performed cardiovascular differentiation using cardiogenic differentiation medium, containing IBM (Iscove’s Basal Medium, Biochrom AG, Berlin, Germany) supplemented with 10% FBS Superior, 1% Cell Shield®, 100 µM non-essential amino acids, 450 µm 1-thioglycerol (Sigma-Aldrich GmbH, Steinheim, Germany), and 213 µg/mL ascorbic acid (Sigma-Aldrich GmbH, Steinheim, Germany). Cardiovascular differentiation was initiated by hanging-drop culture for two days at 37 °C, 5% CO_2_, and 20% O_2_. To start formation of embryoid bodies (EB) 400 cells per drop were plated on the cover of a square petri dish and grown for 2 days. Subsequently, EB grew for four more days in suspension culture and were then harvested for transplantation. These cells will be referred to as cardiac induced cells (CiC) [8].

### Animal model

The experiments in this study were all approved by the federal animal care committee of the Landesamt für Landwirtschaft, Lebensmittelsicherheit und Fischerei Mecklenburg-Vorpommern (*LALLF M-V/TSD/7221.3–1.1–054/15*) and carried out in compliance with the ARRIVE guidelines. Twenty-two 14 – 20 weeks old female 129S6/SvEvTac mice were used for this study and were bred in the animal facility of the Rostock University Medical Center. The mice had free access to water and standard laboratory chow ad libitum and were maintained in specified pathogen-free conditions. For surgery, mice were anesthetized by intraperitoneal administration of pentobarbital sodium (50 mg/kg body weight), subcutaneous injection of fentanyl (0,2 mg/kg) and intubated. Ventilation was ensured using a MiniVent ventilator type 845 (Hugo Sachs Elektronik, March-Hugstetten, Germany). Following thoracotomy, permanent ligation of the left anterior descending coronary artery (LAD) led to myocardial infarction. Afterwards either 1 × 10^6^ CiC suspended in PBS (10 µL) and 10 µL Matrigel™ (MIC group) or only Matrigel™ (MI group) were intramyocardially injected. Four separate injections of 4 × 5 µl were given along the peri-infarct border zone. The injection site was controlled visually at the time of transplantation. Imaging was performed in both groups 17 days (PET/CT) and 21 days (cardiac MRI) following myocardial infarction:

1. MI group (n = 9)
2. MIC group(n = 13)

### PET/CT imaging and analysis

For the PET imaging, mice were anaesthetized by inhalation of isoflurane (2.5% for induction and 1% to 2.5% for maintenance during preparation and scanning). All PET/CT scans were performed on a small animal PET/CT scanner (Inveon MM-PET/CT, Siemens Medical Solutions, Knoxville, TN, USA) according to a standard protocol: A dose of 13.21 MBq ± 4.94 MBq [^18^F]FDG were injected through the mice tail vein via a custom-made micro catheter. After an uptake period of 60 min, mice were imaged in prone position for 20 min. Respiration of the mice was controlled and body temperature was maintained using an infrared heating lamp.

during the imaging procedure.

A Feldkamp algorithm was used to reconstruct CT images. The PET images were reconstructed with the three-dimensional (3D) iterative ordered subset expectation maximization reconstruction algorithm (3D-OSEM/OP-MAP) with the following parameters: 4 iterations (OSEM), 32 iterations (MAP), 1.7 mm target resolution, and 128 × 128 matrix size. Reconstruction included corrections for random coincidences, dead time, attenuation, scatter, and decay [8].

For PET image analysis, PET images were converted from Bq/ml to %ID using PMOD v4.4, and polar maps of the left ventricle were generated with Carimas 2.10 software (Turku PET Centre, Turku, Finland) according to the manual provided by the developer. Results are presented using 17-segmental standardized myocardial segmentation. Metabolic defect size was defined as the area with < 50% of the of the maximum %ID [10].

### Cardiac magnetic resonance imaging and analysis

Cardiac magnetic resonance (CMR) measurements were performed on a 7 Tesla small animal MRI system (BioSpec 70/30, maximum gradient strength 440 mT/m, Bruker BioSpin GmbH, Ettlingen, Germany). The MRI system was equipped with 1H transmit volume coil (86 mm, volume resonator) and a 2-by-2 receive-only surface coil array (both Bruker BioSpin GmbH). Mice were anesthetized using 2% to 3.5% isoflurane in oxygen and placed in a supine position on a dedicated mouse bed and surface coil was placed on the chest of the mice. Respiration rate (35 to 55 breaths per minute) and body temperature were continuously monitored using an MR-compatible small animal monitoring and gating system (Model 1030, SA Instruments, Inc., Stony Brook, NY, USA). Body temperature of the animals was maintained by a water filled heating mat during the whole scan. Monitoring the temperature of the water-filled heating mat was performed by water bath with immersion circulator (HAAKE SC 100 and HAAKE S5P, Thermo Fisher Scientific, Schwerte, Germany). After planning sequences, for the short axes view final images of the left ventricular ejection fraction (LVEF) measurements were acquired using an IntraGate gradient-echo cine sequences (IntraGate Cine-FLASH) in six short-axis planes completely covering the left ventricle. Acquisition parameters included: echo time (TE) 2.38 ms, repetition time (TR) 5.89 ms, flip angle 15°, 14 frames per cardiac cycle, oversampling 140, averages 1, field of view (FOV) 29.4 × 25.2 mm, matrix size 211 × 180, resolution in-plane 0.14 × 0.14 mm, slice thickness 1 mm, and scan time per slice 2 min. Mean heart rates during scanning were 344.8 ± 24.6 bpm in the cell therapy group and 353.0 ± 14.3 in the MI group with no significant difference between the groups (*p* = 0.38).

Cardiac function and morphology were assessed from the cine sequences using the freely available software Segment v4.0 R11044b (http://segment.heiberg.se) as described previously in detail [7]. The primary outcome measure was the LVEF, determined by cardiac MRI.

### Histology

Before the hearts were harvested, a tail vein catheter was placed and 100µg/100 µl biotinylated Lycopersicon esculentum (Tomato lectin) (Vector Laboratories, Burlingame, USA) was injected. Following these procedures hearts were removed and flash frozen in Tissue-Tek® O.C.T. Compound (Sakura Finetek, Alphen aan den Rijn, The Netherlands) with liquid nitrogen.

Frozen hearts were cryo-cut into 6 µm axial sections divided at four different levels from apical to basal. The heart sections were stained with Fast Green FCF (Sigma-Aldrich GmbH, Steinheim, Germany) and Sirius Red (Chroma Waldeck GmbH & Co. KG, Münster, Germany). The relative fibrotic area (Sirius Red positive area/whole left ventricle area) of two contiguous levels was then quantitatively assessed using computerized planimetry (ImageJ/Fiji Software Ver. 2.90) [11].

In order to measure capillary density immunostaining was performed. The sections were fixed in 2% Paraformaldehyd for 10 min at RT. To reduce unspecific binding DAKO Protein and Peroxidase Blocking (Agilent Technologies, Santa Clara, CA, USA) was added. After washing, Tomato Lectin in the endothelial cells was adressed through Goat Anti Biotin Antibody (SP3000, Vector Laboratories, Burlingame, USA). Samples were subsequently incubated for 2 h at 37 °C and stored overnight at 4 °C. The next day samples were incubated with AlexaFluor donkey anti goat secondary antibody (Thermo Fisher Scientific) for 2 h at 37 °C. After washing, the sections were mounted on glass slides with DAKO mounting medium containing DAPI (Agilent Technologies, Santa Clara, CA, USA). Fluorescence images were taken with a Zeiss ELYRA PS.1 LSM 780 confocal imaging system (Carl Zeiss AG). A tissue sample which was not perfused with Tomato Lectin but stained with both antibodies was used as a negative control to verify specificity.

To quantify the capillary number in infarcted scar tissue and uninjured left ventricular myocardium Fiji (Ver. 2.90) was used. In both the infarct and remote area, three randomly selected fields (0.05 mm^2^) were analyzed and the number of capillaries in each field was counted. Subsequently, the average values per 0.05 mm^2^ were determined for the remote and the infarct area to determine capillary density.

### Statistics

All data in this manuscript are presented as mean values ± standard deviation (SD) unless otherwise indicated. Normality of data was assessed by the Shapiro–Wilk test and showed normal distribution. Student’s t-test was used to assess the statistical difference between the groups. Values of p < 0.05 were considered statistically significant. Statistical procedures were performed using SigmaPlot 13.0 (Systat Software, San Jose, CA, USA)*.*

## Results

**Fig. 1:**
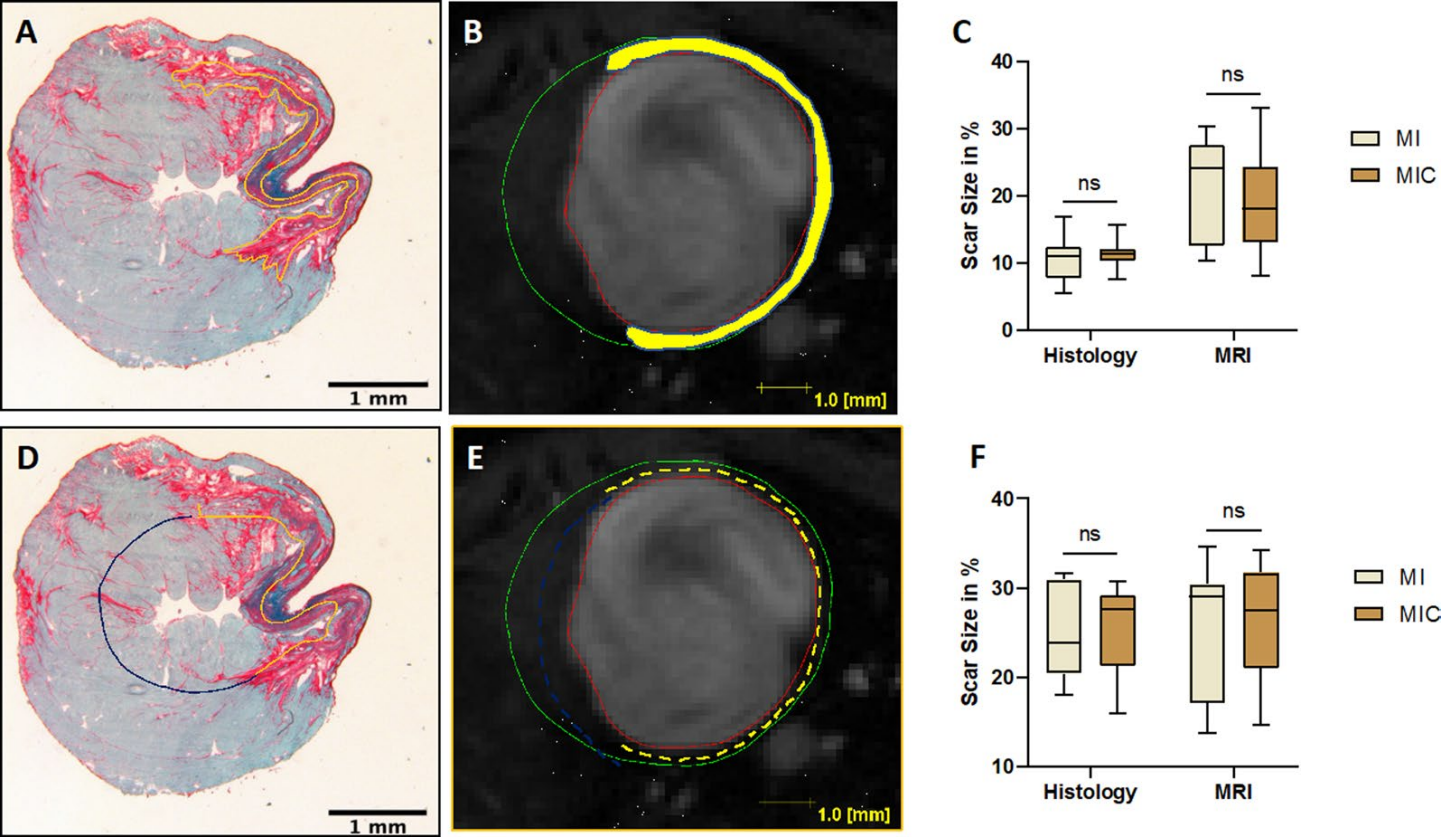
Scar size measurements were performed in both histologic (fast green and sirius red staining) and the respective axial sections of CMR images. **A**–**C** (upper row): Area-based measurement and **C**–**E** (lower row): length-based measurement. 3 weeks after LAD-ligation, there were no differences in scar size between the two groups (MIC: n = 13; MI: n = 9)

**Fig. 2:**
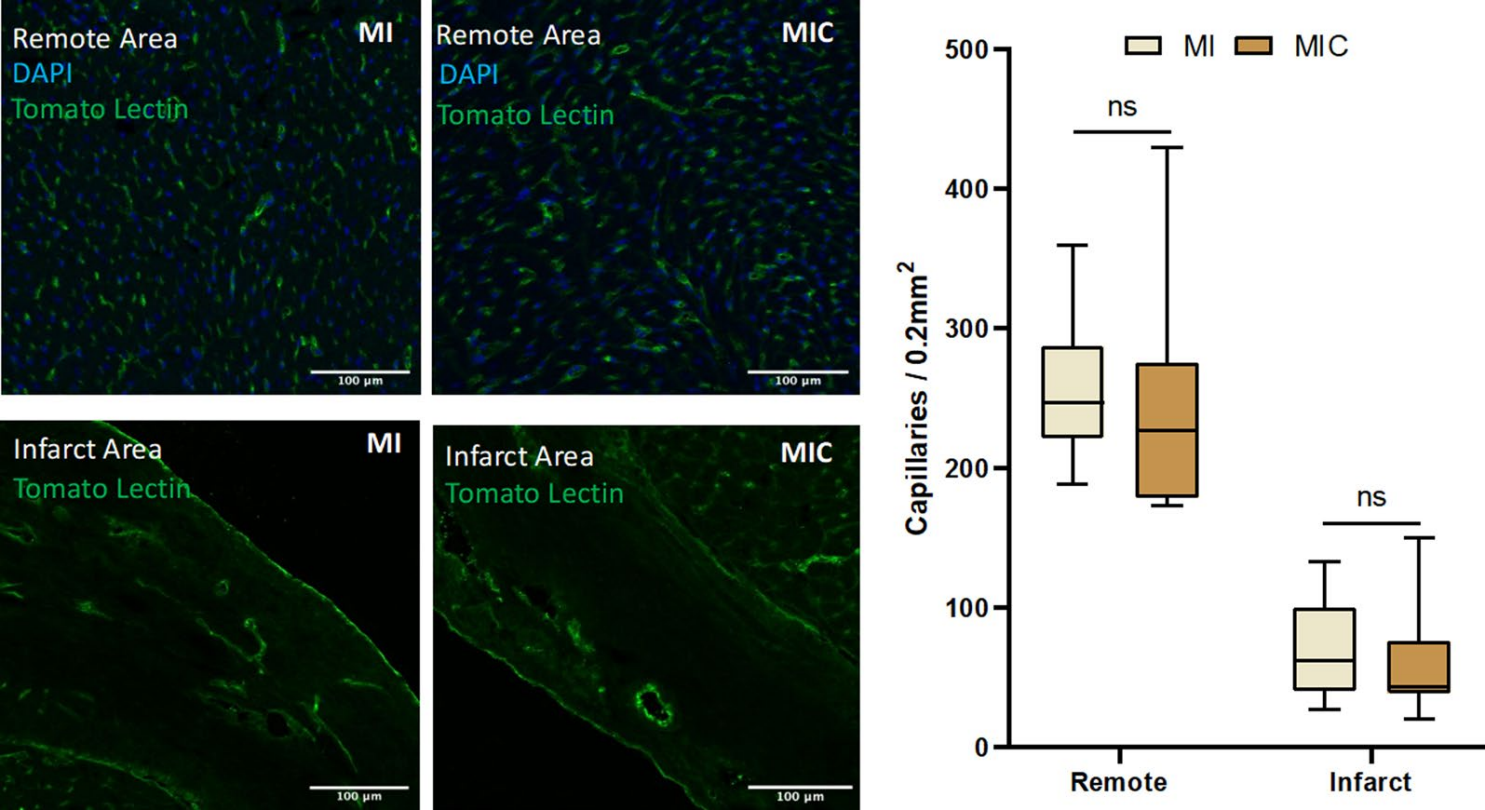
Quantitative assessment of capillary density was performed through fluorescence microscopy using tomato lectin and DAPI staining for cell nuclei. There were no differences in capillary density neither in remote (upper row) nor in infarct area (lower row) between both groups (MIC: n = 13; MI: n = 9)

First, we assessed the effect of cardiac cell therapy on the scar size by both CMR and histology. To enhance the validity of our results, we applied both area and circumference-based approaches for each method (Fig. 1). At 3 weeks after cell transplantation, no statistically significant difference in scar size was detected by either of these methods between control (MI) and therapy group (MIC). (Area-based: histology: 10.4% ± 3.4% vs. 11.4% ± 2.2% (p = 0.42); MRI: 20.4% ± 7.9% vs. 19.3% ± 7.0% (p = 0.73); circumference-based: histology: 25.0% ± 5.2% vs. 25.2% ± 5.0% (p = 0.93); MRI: 24.6% ± 7.7% vs. 26.3% ± 6.1% (p = 0.55)). Next, capillary density, a marker of therapy-induced vessel formation was quantified in both remote and infarcted myocardium. Likewise, no statistically significant differences were detected (Fig. 2).

**Fig. 3:**
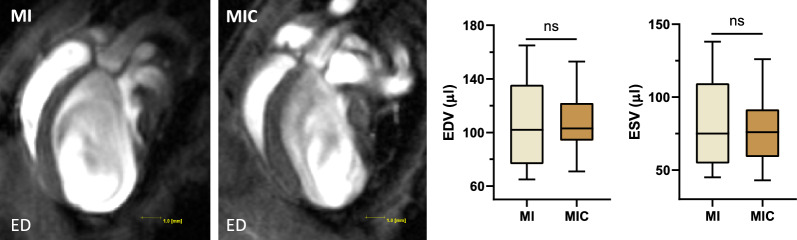
Left Side: Representative end-diastolic CMR images of the left ventricle 3 weeks after myocardial infarction. The long axis view, clearly visualizes the consequences of myocardial infarction: severe dilatation, wall thinning in the infarct area and transition from an elliptical to a more spherical shape of the LV. Right side: No statistically significant differences regarding end-diastolic volume and end-systolic volume could be measured between both experimental groups. (MIC: n = 13; MI: n = 9)

**Fig. 4:**
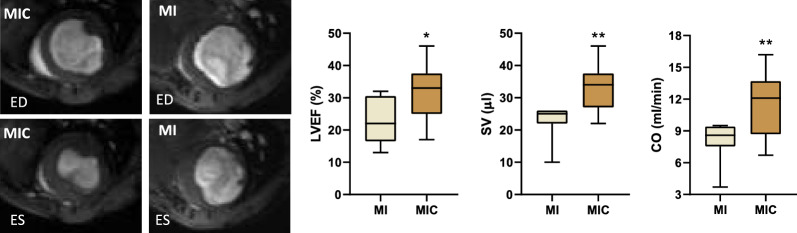
On the left side: representative mid-ventricular short axis CMR images of the control group (MI: n = 9) and cell therapy group (MIC: n = 13) 3 weeks after myocardial infarction. Upper row: end-diastolic (ED). Lower row: end-systolic (ES). On the right side: functional parameters (LVEF, SV and CO) obtained from CMR imaging. * p < 0.05 vs. MI, ** p < 0.01 vs. MI

Next, we measured the effect of cell therapy on LV remodeling, based on the well-established metrics end-diastolic volume (EDV) and end-systolic volume (ESV) [12]. Likewise, no statistically significant differences were detected between control and therapy group (EDV: 106.3µl ± 35.1µl vs. 108.9µl ± 22.0µl (p = 0.84); ESV: 82.8µl ± 32.5µl vs. 76.1µl ± 22.2µl, (p = 0.58)) (Figs. 3 and 4).

### Cell transplantation significantly increased left ventricular performance

Although cell therapy had no effect on global morphological parameters, left ventricular function was significantly improved. In the MIC group, ejection fraction (LVEF), cardiac output (CO) and stroke volume (SV) were significantly increased compared with the MI group (SV: 23.1µl ± 5.3µl vs. 32.9µl ± 7.1µl (p < 0.0024); CO: 8.1ml/min ± 1.9ml/min vs. 11.4 ± 2.9ml/min (p < 0.0074) and LVEF: 23.1% ± 7.0% vs. 31.2% ± 8.5% (p < 0.029)).

### Regional wall properties explain improvement in left ventricular performance

As the improvement of left ventricular performance was apparently not a consequence of reduced global remodeling parameters, we sought to find an explanation based on analyzing regional wall properties of the ventricular wall. Bullseye plots allow both qualitative and quantitative analysis of regional wall thickness and thickening of the left ventricle. Septal (3 and 4) and lateral (1 and 6) segments were analyzed separately.

**Fig. 5:**
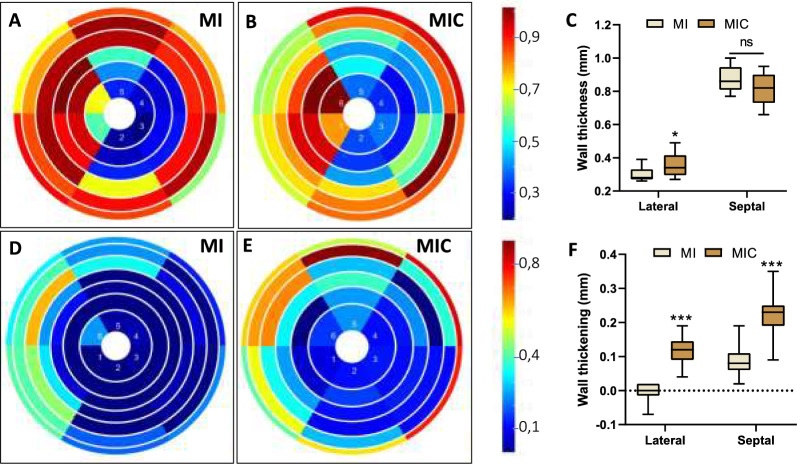
Regional wall thickness **A**–**C** and wall thickening **D**–**F** of the left ventricle 3 weeks after MI visualized as bulls-eye plots. Values are mean ± SD. *p < 0.05 vs. MI, ***p < 0.001 vs. MI (MIC: n = 13; MI: n = 9)

Infarct wall thinning was significantly reduced in the lateral segments after cell therapy (MI vs. MIC: 0.30mm ± 0.05mm vs. 0.36mm ± 0.07mm; p < 0.05) (Fig. 5*)*. Wall thickening, a measure of radial contractility, was significantly improved in both septal and lateral segments in the therapy group (MI vs MIC: septal: 0.09mm ± 0,05mm vs. 0.22mm ± 0.06mm; (p < 0.001); lateral: -0.01mm ± 0.03mm vs. 0.12mm ± 0.04mm; (p < 0.001)) (Fig. 5).

### Cell transplantation significantly reduced glucose metabolic defect as shown via [^18^F]FDG PET imaging

**Fig. 6:**
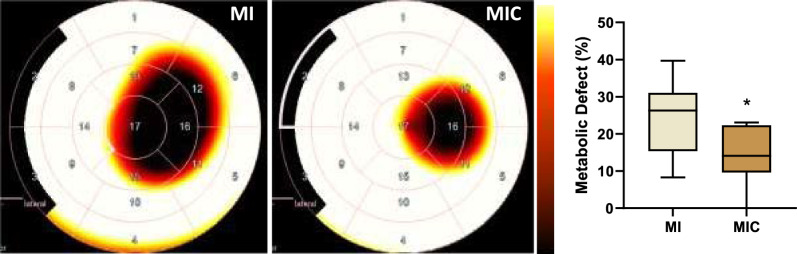
Representative 17-segment tomographic maps visualizing differences of [^18^F]FDG distribution on the left ventricle between the groups. The central segment (segment 17) is equal to the apex. The entire ventricle is mapped 2-dimensionally with the 17-segment model (AHA). Cell therapy significantly reduced the respective metabolic defect size. Scalebar ranges from 20 to 40%ID/ml. *p < 0.05 vs. MI (MIC: n = 8; MI: n = 9)

Based on the finding, that improvement of left ventricular performance following cell transplantation is due to regional wall properties, we hypothesized that regional glucose metabolism might also be affected. Hence, 17 days post-MI, [^18^F]FDG PET/CT was performed to assess the metabolic defect. Cell therapy significantly reduced metabolic defect size after myocardial infarction (14.4% ± 7.9% vs. 23.9% ± 9.9% (p < 0.05)) (Fig. 6).

## Discussion

Left ventricular (LV) remodeling, a typical change in chamber geometry following an acute MI, results in progressive dilation of the ventricle with a consecutive decline of performance. Reducing the extent of LV remodeling is the goal of therapies for the treatment of damaged myocardium. The corresponding metrics are end-diastolic volume (EDV) und end-systolic volume (ESV). The potency of cardiac cell therapies to preserve cardiac function is mostly assessed by measuring the left ventricular ejection fraction (LVEF), which remains the gold standard in evaluating cardiac function [13].

The concept, that transplanted cardiovascular cells replace lost functional myocardium has been abandoned by most researchers and replaced by the concept of the so called “paracrine effects” [2]. The alternative immune response hypothesis has been introduced recently by Molkentin et al*.* [3]. Profound knowledge about the exact mechanisms of action of either concept are still lacking. Yet, the effect of transplanted cells is typically ascribed to distinct phases or elements in the dynamic process of myocardial healing after ischemic injury, such as reduced apoptosis, increased angiogenesis or modulation of the extracellular matrix [14]. The bulk of these biological processes is located within the region of myocardial injury and the adjacent tissue. Against the background that integration of detailed local measures of cardiac function has been proposed to unravel the working mechanisms of cardiac cell therapies [6], we hypothesized that transplantation of cardiovascular progenitor cells improves cardiac function based on improving regional wall properties.

We chose a therapeutic cell preparation we had introduced and characterized recently [8]. In short, murine embryonic stem cells were subjected to a cardiovascular differentiation protocol and transplanted when they had formed stable embryoid bodies. The improvement of LVEF is in the range of results from similar studies [5, 8].

Surprisingly, we found no difference in scar size after 3 weeks, which we assessed by two independent but complementary methods. Furthermore, no effect on capillary density was detected. Both parameters have been commonly used in a plentitude of studies [5]. Yet, a similar study by our group has reported similar findings, with no changes of the fibrotic area and capillary density following transplantation of neonatal cardiomyocytes [15].

Interestingly, we also found no statistically significant difference of global LV dimensions, such as EDV and ESV. Nevertheless, the functional parameters SV, LVEF and CO were statistically significantly improved. Next, regional wall properties were assessed by cardiac MRI, based on a straight-forward protocol we have published recently [7]. As expected, cell therapy resulted in distinct changes of LV wall thickness and radial contractility. Only few studies have assessed regional scar properties after cardiac cell therapy in mice, with similar observations [16–18]. We were surprised by the latter, as evidence from clinical studies suggests that global measures of LV function are inappropriate to detect the relatively small and regional effects of cardiac cell therapies [6]. Integration of detailed local measures of cardiac function have been proposed by experts in field a decade ago, to elucidate the working mechanisms of cardiac cell therapies [6]. Yet, methods for assessment of cardiac function remained surprisingly basic in the vast majority of mouse studies on cardiac cell therapy despite a rapid evolution of dedicated small animal *in-vivo* imaging techniques [5]. Our straight forward approach provides a simple tool for regional wall analysis for the field of research dedicated to improving cardiac function by modifying scar structure and function [19].

## Conclusion

We conclude, that typically used simple methods to assess the effect of cardiac cell therapies, namely scar size and capillary density after formation of the mature scar, are not appropriate metrics to depict the underlying mechanistic effect. Yet, our study clearly shows that regional scar modulation plays a dominant role in the understanding of how a therapy that modulates myocardial healing could rather be assessed. We propose that future studies should address regional scar properties *in-vivo* as described here and to understand the underlying biological processes on a molecular level.

## Data Availability

The datasets used and/or analyzed during the current study are available from the corresponding author on reasonable request.

## Abbreviations

[^18^F]FDG: [^18^F]fluorodeoxyglucose
LVEF: Left ventricular ejection fraction
LV: Left ventricular
CMR: Cardiac magnetic resonance imaging
EB: Embryoid bodies
mESCs: Murine embryonic stem cells
CiC: Cardiac induced cells
LAD: Left anterior descending coronary artery
PBS: Phosphate buffered saline
MI: Myocardial infarction
PET: Positron emission tomography
CT: Computed tomography
SD: Standard deviation
EDV: End-diastolic volume
ESV: End-systolic volume
CO: Cardiac output
SV: Stroke volume
